# Diet and Allergic Diseases among Population Aged 0 to 18 Years: Myth or Reality?

**DOI:** 10.3390/nu5093399

**Published:** 2013-08-29

**Authors:** Danielle Saadeh, Pascale Salameh, Isabelle Baldi, Chantal Raherison

**Affiliations:** 1 Clinical and Epidemiological Research Laboratory, Faculty of Pharmacy, Lebanese University, Beirut 6573-14, Lebanon; E-Mail: pascalesalameh1@hotmail.com; 2 Laboratory “Santé Travail Environnement”, INSERM U897, Institute of Public Health, Epidemiology and Development, University Bordeaux Segalen, Bordeaux 33076, France; E-Mails: isabelle.baldi@isped.u-bordeaux2.fr (I.B.); chantal.raherison@isped.u-bordeaux2.fr (C.R.); 3 Department of Respiratory Diseases, Bordeaux University Hospital, Magellan Avenue, Pessac 33604, France

**Keywords:** diet, dietary patterns, asthma, allergic rhinitis, atopic dermatitis, atopic diseases, children

## Abstract

Allergic diseases are an important health problem. However, epidemiological studies concerning childhood diet-related allergic diseases are scarce. This review examines published articles dealing with diet, dietary patterns and nutrition in relation with allergic diseases among population aged 0 to 18 years. Studies and trials were identified using MEDLINE/PubMed and Cochrane Database of Systematic Reviews and were limited to those published in English or French from 1992 until 2012. This manuscript also reviews the evidence for maternal diet during pregnancy and diet during early childhood and their association with childhood atopic diseases, taking into account the methodology used to evaluate dietary patterns. The evidence reviewed is derived from large epidemiological studies exploring the effects of different food categories on asthma, atopic dermatitis, and allergic rhinitis in children. Overall, maternal diet during pregnancy and a childhood diet rich in antioxidants and omega-3 fatty acids are considered as healthy diets that could be protective for allergic diseases in childhood.

## 1. Introduction

The prevalence of allergic diseases has risen in recent decades, especially among children and in the Western world [1]. This increase in prevalence has become a serious public health issue [2]. Although the etiology of this increase is not clear, it is likely due to a combination of genetic predisposition, environmental factors, and lifestyle changes, including dietary habits [3,4,5,6,7,8,9,10,11].

Pediatric asthma has rapidly increased over the past 20 years, particularly in developed countries, and is the most common chronic inflammatory childhood disease. The symptoms of asthma are dyspnea, wheezing in the chest, and repeated coughing episodes. The prevalence of childhood asthma is 7% in France in 1997 [12,13]. In the United States, six million children (8.5%) have asthma [14]. In general, a family history of allergies is an important indication, directing the diagnosis towards allergic asthma in children [15].

Allergic rhinitis is the inflammation of the nasal mucous membrane that appears after exposure to a particular allergen other than an infection. Although it has received less attention than asthma in epidemiological studies, allergic rhinitis is recognized as the most common allergic manifestation in children and it is more prevalent than asthma [16]. For example, the prevalence of allergic rhinitis among 6–12 year old schoolchildren in Budapest was found to be 14.9% [17].

Atopic dermatitis is a chronic skin disease that affects people with a hereditary history of atopy. Its prevalence has increased considerably in recent decades, especially among children in Western countries, affecting up to 30% of preschool children and 15% to 20% of school-age children [18].

Epidemiological studies on the evolution of atopic diseases, most of which have been cross-sectional and longitudinal, have been conducted over the past decade to determine its possible modifiable determinants [19].

Westernization is associated with an increased prevalence of atopy, allergic rhinitis, and asthma [20]. Several studies have been published on the relationship between diet and childhood allergic diseases such as asthma, allergic rhinitis, and atopic dermatitis or eczema. In general, seven allergens are thought to be responsible for around 90% of food allergy: milk, eggs, wheat, peanuts, nuts, soy, and fish [21]. In contrast, the Mediterranean diet seems protective [22].

This paper examines recently published articles dealing with diet, dietary patterns, and nutrition in association with allergic diseases in children. Moreover, it reviews the evidence for maternal dietary consumption during pregnancy and diet during early childhood, and their association with childhood atopic diseases, taking into account the methodology used to assess dietary patterns.

## 2. Methods

### 2.1. Data Collection

Studies and trials were identified using the following bibliographic databases from the National Institutes of Health (NIH): MEDLINE/PubMed and Cochrane Database of Systematic Reviews [23].

The articles reviewed in this paper were limited to those published in English or French, in the last 20 years (from 1992 till 2012), among populations aged 0 to 18 years.

All records in PubMed were searched using the following terms: “diet” or “nutrition” or “food” or “dietary patterns” or “antioxidants” or “nutrients” or “polyunsaturated fatty acids” or “lipids” or “Mediterranean diet” AND “asthma” or “rhinitis” or “eczema” or “atopy” or “allergy” and “children”.

In addition, we reviewed reference lists of all available primary studies and review articles to identify and consult other potentially relevant citations. These citations were identified using the same bibliographic databases used to search for the primary studies included in this review.

The most recent search was conducted in March, 2012.

### 2.2. Inclusion Criteria

**Figure 1:**
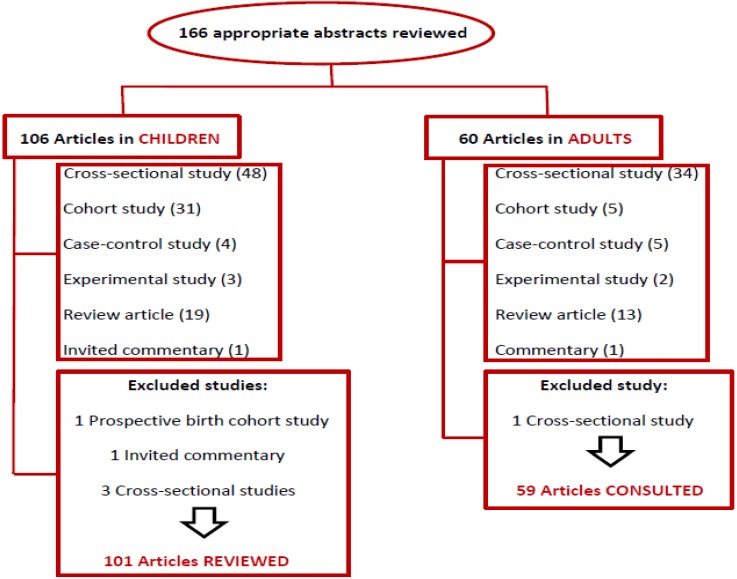
Number of all abstracts consulted, in both adults and children, with the number of excluded and included articles in this review article.

**Figure 2:**
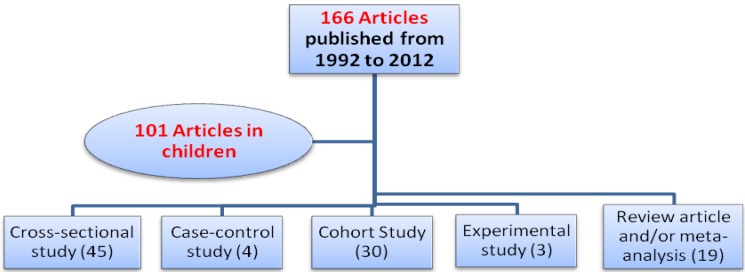
Number and study design of the selected articles reviewed.

We included studies that focused on asthma, allergic rhinitis, and atopic dermatitis as a health outcome related to diet and dietary patterns among population aged 0 to 18 years. We reviewed 101 articles including review articles, cross-sectional, case-control, cohort, and experimental studies (Figure 1, Figure 2).

We included studies that had at least one of the following outcome measures: (1) Lung function measurements (Forced Expiratory Volume in one second (FEV_1_) and/or Forced Expiratory Flow (FEF)); (2) Asthma or asthma symptoms; (3) Wheezing; (4) Rhinitis; (5) Bronchial hyper-responsiveness (BHR); and (6) Eczema or atopic dermatitis.

### 2.3. Exclusion Criteria

Five articles were excluded from the articles reviewed among the population aged 0 to 18 years. One prospective birth cohort study because it was published in 1983, one invited commentary because it was only a commentary and not a full article and three cross sectional studies because after we read the article we found that they were not specifically accurate to our review. Moreover, one cross sectional study was excluded from the adults consulted articles because it was published in 1990 (Figure 1).

## 3. Results and Discussion

### 3.1. Assessment of Diet in Epidemiological Studies

#### 3.1.1. Methodological Issues in the Assessment of Diet

Four methods customarily used in adults were used to assess nutrition or diet in children: (1) 24-h recall, (2) dietary record, (3) the food frequency questionnaire (FFQ), and (4) biomarker measures [24]. The FFQ is the most widely used method because it is applicable to large cohorts and provides information on wide ranges of foods. It assesses long-term diet, it is easy to administer, it allows for repeated measurements so it captures changes in diet over time, and it is relatively cheap to analyze and to calculate nutrients. However, its limitation is that the foods studied need to be validated in a particular population to ensure that the main foods are captured.

Twenty-four-hour recall is a relatively precise method using recent memory but it may not reflect long-term diet like the FFQ and its nutrient analysis may be hard to perform [25]. On the other hand, the dietary record gives a precise record of intake over several days so recall errors and bias are reduced. However, it requires a high level of subject knowledge, literacy, and motivation and may not capture long-term diet [26,27]. Finally, biomarkers are the most precise measure of short-term status and may be the only reliable measurement of nutrient exposure [28,29,30,31]. Unfortunately, they are more expensive and invasive because biological samples such as blood, hair, nail, *etc.*, are collected, so they are less widely used than the other methods in epidemiological studies.

#### 3.1.2. Methodological Issues in Analysis of Dietary Habits

We identified four different approaches used to analyze dietary habits. Most studies used the simple analysis of each nutrient or food category [32]. Some authors used principal component analysis to extract the dietary patterns in their study population. Others used the nutritional profile of the children to create scores like the KIDMED score in the Mediterranean diet, to evaluate and analyze their dietary habits and their adherence to it [23,33,34,35,36]. Moreover, several authors considered the equivalents of food category intake in kilocalories, taking into consideration the metabolic aspect of dietary habits in children and adjusting them to energy intake [27,37,38]. Other authors used more than one approach in evaluating diet in one study [39].

We can never exclude the fact that our results concerning the approaches used for analyzing dietary habits could be biased by the procedure for inclusion of papers in this manuscript and other approaches would have been used by other authors in studies not included in this review. But from our point of view, these four approaches are the most used in epidemiology studies.

In fact, each approach has its own advantages and limitations. In the simple analysis approach, we can evaluate and analysis each food nutrient apart from the other and therefore, present only the results of the significant associations between the nutrient and the health outcome. However, as a limitation for this approach, is that we cannot evaluate the diet of a person as a whole and see its effect on the health outcome. In contrast, when principal component analysis is used, dietary patterns can be extracted and the whole interaction between food nutrients could be associated with the health outcome. Concerning the score creation, it is a very interesting approach to evaluate dietary habits and the adherence to a specific diet. Finally, the use of the equivalents of food nutrients intake and energy intake is a more specific approach to evaluate each nutrients by itself in a more precise dose than the simple analysis approach, but again it cannot evaluate all nutrients composing a diet all-together.

Often, authors choose the most suitable and valid approach, score or method to assess dietary habits and to present their results and some authors use more than one approach in the same study to include more precisely all associations that could explain their objectives.

### 3.2. Results and Association between Diet and Atopic Diseases in Recent Childhood Studies

In this section, we report the most important findings with regard to dietary factors and allergic diseases in youths, in recent epidemiological studies performed worldwide (Table 1). The associations shown in these studies are commented, citing the advantages and disadvantages of each methodology.

**Table 1:** Results and association between diet and allergic diseases in children in recent international epidemiological studies.

Reference; Country; Study Type	Population	Diet Assessment	Dietary Evaluation	Health Outcome	*p*-value	aOR	95% CI
Arvaniti *et al.*, (2011) [35]; Greece; Cross-sectional	700 children, 10–12 years	FFQ and KIDMED score	Adherence to the Mediterranean Diet	Ever had wheezing	0.001		
				Exercise wheezing	0.004		
				Night cough	0.07		
				Ever had diagnosed asthma	0.002		
				Any asthma symptoms	<0.001		
			KIDMED score	Asthma symptoms	<0.001	0.86	0.75–0.98
Martindale *et al.*, (2005) [40]; UK; Birth cohort	1924 children followed up to 2 years of age	FFQ during pregnancy at 34 weeks gestation	Maternal vitamin E intake	Wheezing in the absence of a “cold” in the second year of life	0.009	0.49	0.26–0.93
				Eczema	0.016	0.42	0.22–0.82
			Maternal vitamin C intake	Ever wheezing	0.01	3	1.47–6.12
				Eczema	0.048	1.56	0.99–2.45
Oh *et al.* (2010) [30]; Korea; Case-Control	180 AD and 242 non-AD preschoolers	FFQ and blood samples for fat-soluble vitamins (retinol, α-tocopherol, and β-carotene) and vitamin C	β-carotene	Atopic dermatitis	0.03	0.44	0.22–0.88
			Vitamin E		<0.001	0.33	0.16–0.67
			Folic acid		<0.001	0.37	0.18–0.73
			Iron		0.01	0.39	0.19–0.79
			α-tocopherol		0.037	0.64	0.41–0.98
			Retinol (vitamin A)		0.022	0.74	0.58–0.96
			Vitamin C		0.559	0.94	0.76–1.17
Hijazi *et al.*, (2000) [41]; Saudi Arabia; Case-Control	114 cases and 202 controls	FFQ and SPTs	Fast food	Asthma and wheezing	0.008		
			Animal fat for cooking		0.062		
			Eating fish		0.073		
			Milk daily		0.04	2.4	1.21–4.75
			Vegetables		0.01	2.83	0.98–8.09
			Fiber		0.009		
			Vitamin E		0.005	3	1.38–6.50
			Magnesium		0.001		
			Calcium		<0.001		
			Sodium		<0.001	2.88	1.42–5.87
			Potassium		0.002		
Miyake *et al.*, (2010) [42]; Japan; Birth cohort	763 mother-child pairs	Diet history questionnaire 16-days dietary records	Total vegetables	Wheezing	0.23	0.69	0.41–1.15
			Total fruit		0.11	1.51	0.90–2.57
			Total vegetables	Eczema	0.22	0.7	0.41–1.19
			Green and yellow vegetables		0.01	0.41	0.24–0.71
			Total fruit		0.34	0.78	0.45–1.35
			Citrus fruit		0.03	0.53	0.30–0.93
			Vitamin E	Wheezing	0.04	0.54	0.32–0.90
			Zinc		0.06	0.69	0.41–1.17
			β-carotene	Eczema	0.04	0.52	0.30–0.89
			Vitamin E		0.15	0.59	0.34–1.02
Palmer *et al.*, (2012) [43]; South Australia; Randomized controlled trial	706 infants at high hereditary risk of having allergic disease. Intervention group (*n* = 368); Control group (*n* = 338)	Ig E associated allergic disease at 1 year of age; SPTs	*n*-3 (LCPUFA)	Egg sensitization	0.02	0.62	0.41–0.93
			*n*-3 LCPUFA	Eczema with sensitization	0.06	0.64	0.40–1.03
Nwaru *et al.*, (2011) [44]; Finland; Birth cohort study with 5-year follow-up	2441 children	Maternal FFQ data	Magnesium	Eczema		0.78	0.62–0.97
Miyake *et al.*, (2011) [45]; Japan; Cross-sectional	23,388 schoolchildren 6–15 years	Diet history questionnaire for children and adolescents	PUFA	Eczema	0.04	1.26	1.07–1.48
			*n*-3 Long Chain PUFA		0.009	1.31	1.11–1.54
			α-linoleic acid		0.003	1.31	1.12–1.55
			*n*-6 PUFAs		0.01	1.26	1.07–1.48
			Linoleic acid		0.01	1.27	1.08–1.49
			Arachidonic acid	Eczema	<0.001	0.81	0.69–0.95
			Arachidonic acid	Rhinoconjunctivitis	0.03	0.86	0.74–0.99
Emmanouil *et al.*, (2010) [26]; Greece; Cross-sectional study	1964 children, 24–72 months	3 days diet records	Vitamin C	Ever wheeze		0.997	0.99–1.00
			Vitamin C	Current wheezing		0.996	0.99–0.99
			Calcium	Current wheezing		0.999	0.99–0.99
			Magnesium	Current wheezing		1.005	1.00–1.00
			MUFA	Ever wheezing		1.023	1.00–1.04
			MUFA	Current wheezing		1.02	1.00–1.03
			Magnesium	Diagnosed asthma		1.006	1.00–1.01
Miyake *et al.*, (2010) [46]; Japan; Birth cohort	763 mother-child (16–24 months) pairs	Diet history questionnaire during pregnancy	Dairy products	Wheezing	0.007	0.45	0.25–0.79
			Milk		0.02	0.5	0.28–0.87
			Cheese		0.02	0.51	0.31–0.85
			Calcium		0.04	0.57	0.32–0.99
			Vitamin D	Wheezing		0.64	0.43–0.97
			Vitamin D	Eczema		0.63	0.41–0.98
Suárez-Varela *et al.*, (2010) [23]; Spain; Cross-sectional	13,153 schoolchildren 6–7 years	FFQ and a Mediterranean diet score	Butter	Atopic Dermatitis	0.04	0.7	0.50–0.97
			Nuts		0.003	0.51	0.33–0.80
			Milk (once or twice a week)	Atopic Dermatitis	0.007	0.42	0.22–0.79
			Milk (3 or more times a week)		0.001	0.5	0.33–0.75
			Mediterranean diet score		0.071	1.03	0.99–1.08
Nagel *et al.*, (2010) [47]; 29 centers in 20 countries; Multicenter cross-sectional studies	50,004 schoolchildren 8–12 years, (29,579 with skin prick testing)	FFQ and a Mediterranean diet score	Fruit intake	Current wheezing in affluent countries	0.168	0.86	0.73–1.02
			Fruit intake	Current wheezing in non-affluent countries	0.168	0.71	0.57–0.88
			Fish	Asthma ever	0.04	0.92	0.78–1.08
			Cooked green vegetables	Current wheezing in non-affluent countries	0.018	0.78	0.65–0.95
			Burger consumption	Current wheezing	0.05	1.12	0.86–1.45
			Meat intake	Asthma ever	0.03	1.11	0.90–1.35

aOR: Adjusted Odds Ratio; CI: Confidence Interval; FFQ: Food Frequency Questionnaire; KIDMED: Score for adherence to the Mediterranean diet in children; SPTs: Skin Prick Tests; IgE: Immunoglobulin E; LCPUFA: Long Chain Polyunsaturated Fatty Acids; PUFA: Polyunsaturated Fatty Acids; MUFA: Monounsaturated Fatty Acids.

#### 3.2.1. The Mediterranean Diet

Studies on adherence to the Mediterranean diet are numerous. In fact, it is now considered as a healthy diet and has been proven as a protective factor against allergic diseases in most of the studies performed in children.

We reviewed five cross-sectional studies and one review article which all found that the adherence to the Mediterranean diet was inversely related with atopy in children (Table 2). Adherence to the Mediterranean diet, and following a healthy dietary pattern by eating more fruits and vegetables, for example, has been shown to be a protective factor for atopic diseases in children in many countries such as Sweden, Greece, and Mexico [39,48,49]. In addition, a high level of adherence to the Mediterranean diet was a protective factor for current wheezing in children [47,50]. One study found no association between the Mediterranean diet and atopic dermatitis (adjusted odds ratio (aOR) = 1.03, 95% confidence interval (CI) = 0.99–1.08) [22].

In Athens, Arvaniti *et al.* studied adherence to the Mediterranean diet in children aged 10–12 years in relation to wheezing and asthma symptoms. They found that a high level of adherence to the Mediterranean diet had a protective effect on asthma symptoms (aOR = 0.86; 95% CI = 0.75–0.98) [35]. In this study, dietary habits were evaluated by an FFQ completed by the children and their parents through face-to-face interviews, and a special diet score, the KIDMED score, was calculated to evaluate adherence to the Mediterranean diet. Higher KIDMED scores corresponded to greater adherence to the diet. There was a lack of association between single foods and asthma prevalence, probably due to a recall bias because information about the diet was provided by the parents, but the overall results suggested a protective effect of a high level of adherence to the Mediterranean diet on asthma symptoms.

Although there have been several nutritional studies assessing single nutrients instead of assessing dietary patterns, the single food approach may fail to account for interactions between nutrients. Indeed, people eat meals consisting of a variety of foods with combined nutrients, not just isolated foods [51]. Furthermore, Nagel *et al.* studied food categories in relation with asthma and wheezing in a multicenter study (ISAAC phase 2) of more than 50,000 schoolchildren aged 8–12 years old. Fruit intake was associated with a low prevalence of current wheezing in both affluent and non-affluent countries (*p* = 0.168). Consumption of fish in affluent countries (aOR = 0.92, 95% CI =0.78–1.08) and of cooked green vegetables in non-affluent countries (aOR = 0.78, 95% CI = 0.65–0.95) was associated with a lower prevalence of current wheezing and asthma [47].

An FFQ was used to collect information retrospectively by parents about the usual diet consumed by their children and then a Mediterranean dietary pattern was derived according to the consumption frequency of food items. The data collected retrospectively may have also introduced a recall bias, but it gave the exact recent consumption. Moreover, the authors in this study could not adjust for total energy intake and body mass index because it was based on the ISAAC questionnaire [35].

Despite the limitations of the cited studies, the consistency of the findings pleads in favor of the inverse relationship between the Mediterranean diet and allergic diseases.

#### 3.2.2. Antioxidant Nutrient Intake

Evidence is accruing of the protective effect of antioxidants and vitamins for health problems such as asthma, allergic rhinitis, and atopic dermatitis. Our knowledge of the health effects of these antioxidant nutrients is far from complete owing to the inconsistent or even contradictory epidemiological studies undertaken on this subject, especially in children.

We reviewed a total of 13 articles in relation to fruit and vegetable consumption, antioxidant, and vitamin intake, in the association with health outcomes such as allergic diseases, atopy, lung function levels and respiratory symptoms, childhood asthma, asthma symptoms, wheezing, atopic dermatitis or eczema, allergic sensitization, and allergic rhinitis. The majority of these studies were cross-sectional (*n* = 8), three were birth cohort studies, and there was one case-control study on atopic dermatitis, and one review article (Table 2). In general, antioxidant intake including fruit, vegetables, and vitamins may be considered as protective against allergic diseases in children.

**Table 2:** Reviewed studies on antioxidant nutrients, vitamins, and Mediterranean diet in children and adolescents and their association with allergic diseases.

Reference; Country; Study Type	Population	Significant Food Association	Health Outcome Measured	Association
Cook *et al.*, 1997 [52]; UK; Cross-sectional	2650 children	Fresh fruit (vitamin C)	Lung function (FEV_1_) and wheezing	Fresh fruit intake was associated positively with lung function.
Forastiere *et al.*, 2000 [53]; Italy; Cross-sectional	18,737 children, 6–7 years	Fruit rich in vitamin C	Wheezing symptoms	Consumption of fruit rich in vitamin C may reduce wheezing symptoms in childhood.
Gilliland *et al.*, 2003 [54]; California; Cross-sectional	2566 children	Dietary antioxidants and vitamin A	Lung function levels (FEV_1_ and FEF_25–75_)	An inadequate dietary antioxidant vitamin intake is associated with reduce lung function levels in children.
Kalantar-Zadeh *et al.*, 2004 [55]; Review article	In children	Dietary antioxidants	Childhood asthma	Most studies indicate a higher prevalence of dietary antioxidant deficiency among asthmatic patients; But results seem to be contradictory; More epidemiological studies are needed.
Rubin *et al.*, 2004 [56]; New York; Cross-sectional	7505 children, 4–16 years	Serum antioxidants	Childhood asthma	No association of vitamin E with asthma; High β-carotene, vitamin C and selenium intake was associated with a reduction in asthma.
Harik-Khan *et al.*, 2004 [57]; New York; Cross-sectional	4,093 children, 6–17 years	Vitamin C and α-carotene	Asthma	Low vitamin C and α-carotene intake are associated with asthma risk.
Chatzi *et al.*, 2007 [33]; Greece; Cross-sectional	690 children, 7–18 years	Mediterranean diet	Allergic rhinitis, asthma and atopy	High dietary intake of fruits, vegetables and nuts may have a protective role on the prevalence of asthma symptoms and allergic rhinitis.
Okoko *et al.*, 2007 [58]; UK; Cross-sectional	2560 children, 5–10 years	Fruits	Asthma, asthma symptoms and wheezing	Banana consumption and drinking apple juice were negatively associated with wheezing but not asthma.
Chatzi *et al.*, 2007 [59]; Spain; Cross-sectional	460 school children, 6.5 years	Fruits and vegetables	Wheezing, atopic wheezing and atopy	Fruit and vegetable intake was inversely associated with current and atopic wheezing.
Burns *et al.*, 2007 [60]; United States and Canada; Cross-sectional	2112 students, 16–19 years	Fruits, antioxidants, nutrients and *n*-3 fatty acids	Pulmonary function and respiratory symptoms	Adolescents with the lowest dietary intake of antioxidant had lower pulmonary function and increased respiratory symptoms, especially among smokers.
De Batlle *et al.*, 2008 [49]; Mexico; Cross-sectional	1476 children aged 6–7 years old	Mediterranean diet	Asthma and rhinitis	Mediterranean diet has a protective effect on asthma and allergic rhinitis in children.
Castro-Rodriguez *et al.*, 2008 [50]; Spain; Cross-sectional	1784 preschoolers4 years old	Mediterranean diet	Current wheezing	Mediterranean diet was shown as a protective factor for current wheezing.
Bacopoulou *et al.*, 2009 [61]; Greece; Birth cohort (18 years follow-up)	2133 children, 7- and 18- years	Fruits and vegetables	Asthma	Daily consumption of fruits and vegetables was negatively associated with current asthma.
Patel *et al.*, 2009 [62]; UK; Birth cohort (8 years follow-up)	861 children, 5- and 8-years	Dietary antioxidant	Wheezing or eczema, allergic sensitization and immunoglobulin E levels	No association between antioxidant intakes and wheezing or eczema; Increased beta-carotene intake was associated with a reduced risk of allergic sensitization and lower immunoglobulin E levels.
Chatzi and Kogevinas, 2009 [63]; Review article	In children	Mediterranean diet	Asthma and atopy	High level of adherence to Mediterranean diet in early life protects against development of asthma and atopy in children.
Oh *et al.*, 2010 [30]; Korea; Case-control	Children, 5–6 years (180 cases and 242 controls)	Antioxidant nutrients	Atopic dermatitis	Higher antioxidant intake reduces risk of atopic dermatitis (AD); No relationship of AD with vitamin C.
Nagel *et al.*, 2010 [47]; in 20 countries; Cross-sectional	50,004 school children, 8–12 years	Mediterranean diet	Wheeze and asthma	Adherence to Mediterranean diet may provide protection against wheeze and asthma.
Gonzalez Barcala *et al.*, 2010 [36]; Spain; Cross-sectional	14,700 children and adolescents	Mediterranean diet	Asthma	Greater adherence to the Mediterranean Diet (MD) is associated with a higher risk of severe asthma in girls of 6–7 years; The results of the study do not support a protective effect of MD on prevalence or severity of asthma.
Rosenlund *et al.*, 2011 [39]; Sweden; Birth cohort (8 years follow-up)	2447 children	Fruit intake	Allergic disease	Inverse association between fruit intake and allergic disease in children.

FEV_1_: Force expiratory volume in 1second; FEF_25–75_: Forced expiratory flow between 25% and 75% of forced vital capacity.

##### 3.2.2.1. Fruit and Vegetables

A diet high in antioxidants may prevent the expression of allergic diseases. Furthermore, a diet rich in fruit, vegetables and antioxidants may contribute to optimal respiratory health [33,59,60].

Hijazi *et al.* suggested that lower intake of vegetables (aOR = 2.83, 95% CI = 0.98–8.09) was negatively associated with asthma [41]. In fact, an FFQ was used in this study performed in Saudi Arabia that included the main foods present in the Saudi diet, *i.e.*, vegetables and local fruit, such as dates. These results provide information about the dietary intake in Arab groups rather individuals so the food studied need to be validated in this particular population to ensure that the main foods are captured.

Forastiere *et al.* concluded that the consumption of fruit rich in vitamin C may reduce wheezing symptoms in childhood [53]. This could be due to the fact that vitamin C may protect airways against oxidant attack [64], thus reducing asthma symptoms. An information bias is possible in this study, since a parental questionnaire was used to evaluate both dietary factors and health outcomes. However, questionnaire data on dietary intake completed by parents are generally considered to be reliable, especially regarding vitamin C intake [65]. Moreover, an infrequent consumption of fresh fruit may be an indicator of a poor diet, which may lack vitamins other than vitamin C, such as A and E, that may also have a protective effect on respiratory symptoms [64,66]. Intake of vitamins C and E is usually correlated [67] and both are likely to play a synergistic role in the antioxidant defense mechanism [64].

In contrast, Cook and coworkers found no association between vitamin C levels in fresh fruit and lung function in children [52]. The lack of statistical significance in that study could be due to the small number of subjects with symptoms. The lack of a relationship between wheezing and fresh fruit consumption could be due to protection against bronchoconstriction in susceptible individuals, which is in accordance with a recent study in adults [68]. Moreover, Oh *et al.* found no relationship between atopic dermatitis risk and plasma vitamin C (aOR = 0.94, 95% CI = 0.76–1.17) [30].

In Greece, Emmanouil *et al.* found that vitamin C intake was negatively associated with the prevalence of ever wheezing (aOR = 0.99, 95% CI = 0.99–1.00) and current wheezing (aOR = 0.99, 95% CI = 0.99–0.99) in children [26]. Dietary intake was assessed with a three-day dietary record. Therefore, over- or under-reporting of dietary intake may have confounded the results. Another limitation of this study was that it focused on isolated nutrients by using a simple analysis of each nutrient and did not take into account food groups or specific food items.

##### 3.2.2.2. Dairy Products

Regarding the consumption of dairy products, including milk consumption, we reviewed three articles: two cross-sectional studies and one case-control study. These studies suggested that milk consumption could be associated with a protective effect on atopic eczema and asthma symptoms.

In Spain, Suárez-Varela *et al.* used a FFQ and a Mediterranean diet score to show that milk was negatively associated with atopic dermatitis in children aged six to seven years old, if consumed once or twice a week (aOR = 0.42, 95% CI = 0.22–0.79) [22]. In fact, milk is a source of saturated fats that has been associated with a protective effect on atopic disease [69].

In Saudi Arabia, Hijazi *et al.* suggested that a lower consumption of milk (aOR = 2.4, 95% CI = 1.21–4.75) is associated with a higher prevalence of asthma symptoms and allergy [41].

##### 3.2.2.3. Other Vitamins and Nutrients

The total articles reviewed with their study designs regarding vitamins and nutrients are cited in the beginning of the section “Antioxidant nutrient intake”.

In Korea, Oh *et al.* showed in a case-control study that atopic dermatitis was negatively associated with intake of antioxidant nutrients [β-carotene (aOR = 0.44, 95% CI = 0.22–0.88), vitamin E (aOR = 0.33, 95% CI = 0.16–0.67), folic acid (aOR = 0.37, 95% CI = 0.18–0.73), and iron (aOR = 0.39, 95% CI = 0.19–0.79)]. Reduced atopic dermatitis risk was found with the increase in α-tocopherol (aOR = 0.64, 95% CI = 0.41–0.98) and retinol concentrations (aOR = 0.74, 95% CI = 0.58–0.96) [30].

Diet was assessed using a validated semi-quantitative FFQ. Fasting blood samples were used to analyze fat-soluble vitamins (retinol, α-tocopherol, and β-carotene) and vitamin C. The authors assessed antioxidant nutrient intake by FFQ in the previous 12 months, which provided long-term regular intake. In contrast, serum antioxidant nutrients are indices of short-term nutritional status [70]. These findings suggest that long-term antioxidant nutritional status measures would be more sensitive markers for atopic dermatitis than recent ones. Furthermore, the unavailability of supplementary intake of vitamins and minerals in this study may have underestimated the true association between some nutrients and atopic dermatitis.

In Finland, Nwaru *et al.* showed that dietary intake of magnesium was negatively associated with eczema in children (aOR = 0.78, 95% CI = 0.62–0.97) [44]. An FFQ for maternal intake was used but it had the same limitations as every FFQ, *i.e.*, it has to be used in a specific population, in this study, the Finnish population, and the food studied needs to be validated in that population to ensure that the main foods are captured.

In Saudi Arabia, Hijazi *et al.* suggested that fiber (*p* = 0.009), vitamin E (aOR = 3, 95% CI= 1.38–6.50), magnesium (*p* < 0.001), calcium (*p* < 0.001), sodium (aOR = 2.88, 95% CI = 1.42–5.87), and potassium (*p* = 0.002) were negatively associated with asthma after adjustment for energy intake [41]. In this study, diet and risk factors for allergies were evaluated by the ISAAC questionnaire translated into Arabic and atopy was assessed by skin prick testing. The FFQ may provide useful information on intake only in groups rather than individuals and may be used for comparisons between these groups. Moreover, the FFQ used in this study may be an inappropriate method to demonstrate the intake of some nutrients like salt because of the geographical setting of the Saudi Arabia compared to western countries.

#### 3.2.3. Fat and Fish Studies

Fat and fish intake have been correlated with allergic diseases in children. We reviewed a total of 11 articles that studied the relationship between fish consumption and fat intake and allergic diseases in children. The majority of these studies were cross-sectional (*n* = 8), in addition to one cohort study, one nested case-control study, and one randomized controlled trial (Table 3). Epidemiological studies have shown that fish intake, monosaturated fats and omega-3 polyunsaturated fatty acids have a protective effect on asthma, respiratory health, and atopic dermatitis [28,71,72,73,74]. Other studies were contradictory and showed that *n*-3 and *n*-6 polyunsaturated fatty acids may be risk factors for asthma symptoms and eczema [45,75]. On the other hand, fatty foods of animal origin and saturated fatty acids were a risk factor for asthma in children [27,38].

**Table 3:** Reviewed studies on fish and fat intake in infants and children and their association with allergic diseases.

Reference; Country; Type of Study	Population	Significant Food Association	Health Outcome Measured	Association
Peat *et al.*, 1992 [73]; Australia; Cross-sectional	4366 children	Fish	Bronchial hyper responsiveness	Protective effect of fish on bronchial hyper responsiveness in children.
Hodge *et al.*, 1996 [72]; Australia; Cross-sectional	574 children	Oily fish	Asthma	Consumption of oily fish may protect against asthma in childhood.
Yu *et al.*, 1996 [28]; Sweden; Birth cohort	68 infants	*n*-3 and *n*-6 fatty acids	Allergic dermatitis and asthma	Significant correlations found between *n*-3 and *n*-6 fatty acids in the cord blood of children who did not develop allergic dermatitis or asthma by 6 years of age.
Huang *et al.*, 2001 [38]; Taiwan; Cross-sectional	1166 children, 13–17 years	Fat-rich foods; saturated fats and monounsaturated fats	Asthma	A higher prevalence of asthma was related to fat-rich foods of animal origin; Saturated fats were associated with increased risk of asthma. Monounsaturated fats were inversely associated with asthma.
Takemura *et al.*, 2002 [76]; Japan; Cross-sectional	Schoolchildren, 6–15 years	Fish	Asthma	Higher fish intake was positively related to prevalence of asthma.
Antova *et al.*, 2003 [74]; Six European countries; Cross-sectional	20,271 children, 7–11 years	Fish	Respiratory health	Low fish intake was a consistent predictor of poor respiratory health.
Murray *et al.*, 2006 [77]; England; Nested case-control study	541 children, 3 years old	Polyunsaturated fat	Atopy and wheezing	Sensitized wheezy children had a higher total polyunsaturated fat intake compared with non-sensitized non-wheezy children.
Miyake *et al.*, 2008 [75]; Japan; Cross-sectional	25,033 children, 6–15 years	Fatty acids	Asthma symptoms and wheezing	Both *n*-3 and *n*-6 polyunsaturated fatty acids may be associated with an increased prevalence of wheezing.
Al Biltagi *et al.*, 2009 [71]; Egypt; Randomized Controlled Trial	60 children, 7–10 years	Omega-3 fatty acids	Asthma and pulmonary function	Diet supplementation with omega-3 fatty acids significantly improved asthma control and pulmonary functions.
Rodri’guez-Rodri’guez *et al.*, 2010 [27]; Spain; Cross-sectional	638 school children, 8–13 years	Saturated fatty acids (SFAs)	Current asthma	Increased intakes of SFAs, especially butter, seem to be related to current asthma.
Miyake *et al.*, 2011 [45]; Japan; Cross-sectional	23,388 schoolchildren, 6–15 years	*n*-3 and *n*-6 PUFAs	Eczema and rhino conjunctivitis	Intake of *n*-3 and *n*-6 PUFAs may be positively associated with eczema; Arachidonic acid intake may be inversely related to eczema and rhino conjunctivitis.

PUFAs: Polyunsaturated Fatty Acids.

##### 3.2.3.1. Fat Intake

Several studies have shown that diet supplementation with omega-3 polyunsaturated fatty acids significantly improved pulmonary function tests, thus childhood asthma, as well as having a protective effect against allergic dermatitis [28,71]. On the other hand, increased consumption of *n*-6 polyunsaturated fatty acids (PUFAs) reportedly contributed to the recent increased prevalence of asthma and may be associated with an increased prevalence of wheezing in children [75].

A more recent study by Miyake *et al.* in Japan on more than 23,000 schoolchildren 6–15 years old showed that consumption of both *n*-3 and *n*-6 polyunsaturated fatty acids was positively associated with the prevalence of eczema (aOR = 1.31, 95% CI = 1.11–1.54 and aOR = 1.26, 95% CI = 1.07–1.48, respectively). In contrast, polyunsaturated omega-6 fatty acid (arachidonic acid) may be negatively associated with eczema (aOR = 0.81, 95% CI = 0.69–0.95) and rhino-conjunctivitis (aOR = 0.86, 95% CI = 0.74–0.997) [70]. Both *n*-3 and *n*-6 polyunsaturated fatty acids may be associated with an increased prevalence of wheezing [75]. A self-administered diet history questionnaire was used to assess children’s dietary intakes in the Miyake *et al.* study [45]. This method should be interpreted with caution as it might not reflect long-term intake by children and may underestimate the results. Moreover, the authors did not include intake of dietary supplements in this study because their use is uncommon in Japan.

In South Australia, Palmer *et al.* showed in their randomized controlled trial (RCT) that *n*-3 long chain polyunsaturated fatty acid supplementation in pregnancy did not reduce the overall incidence of immunoglobulin E-associated allergies in the first year of life, although atopic eczema was decreased (aOR = 0.64, 95% CI = 0.40–1.03) [43]. In this RCT, the concentration of long chain polyunsaturated fatty acids (LCPUFA) in plasma phospholipids from cord blood was evaluated as an independent biomarker of adherence to the protocol supplementation of the women in the *n*-3 LCPUFA and in the control group. The calculation of the concentration of LCPUFA in this RCT is a precise measure of short-term status and may be the only reliable measurement of LCPUFA intake.

##### 3.2.3.2. Fish Intake

Concerning fish intake, results of studies in children seem contradictory. Consumption of oily fish may protect against asthma in childhood [72] and eating fish more than once a week has a protective effect on bronchial hyper-responsiveness and asthma in children [73,78]. On the other hand, frequency of fish intake was positively related to the prevalence of asthma in a cross-sectional study done in Japan [78]. In contrast, Hijazi *et al.* in Saudi Arabian study suggested that eating fish (*p* = 0.073) was not significantly related to asthma and wheezing [41]. These discrepancies could be explained by the differences in diet between countries. In fact, the Japanese often eat fish cooked with salt, which could lead to the positive association between fish and asthma in this study [78]. In contrast, the absence of relationship observed between fish intake and asthma in the Saudi population could be due to their lower consumption of fish and frequent consumption of lamb and chicken.

#### 3.2.4. Fast Food and Soft Drinks

Fast food consumption is considered unhealthy especially for children. It contributes to increasing obesity and can also increase the prevalence of chronic diseases like asthma in children [79]. We reviewed six articles regarding fast food and/or soft drinks consumption and allergic diseases in childhood: one case-control study and five cross-sectional studies. Recently, respiratory allergic diseases in children such as asthma, wheezing and bronchial hyper-responsiveness have been correlated with the high consumption of fast food, like the frequent consumption of hamburgers, salty-snack eating, and frequent takeaway consumption [41,80]. These associations were more significant in children with a higher sedentary lifestyle such as watching television or playing video games for more than two hours a day [81]. An FFQ was completed by parents in the latter study, so the results could be subject to recall bias and the behaviors of the children could be either under- or over-estimated. In fact, a child who is already asthmatic may change his behaviors by staying indoors more often in order to avoid outdoor allergens. Therefore, he will increase his television-watching and will lead a more sedentary life, which in turn may lead to obesity and increase asthma symptoms [82].

Moreover, the consumption of soft drinks containing preservatives or colorants increased significantly the risk of allergic rhinitis symptoms in children [17]. All these studies used the FFQ to evaluate fast food intake [17,41,79,80,81,82]. Future research into the effects of diet on allergic disease should focus on these unhealthy foods whose consumption has increased in recent years, and include further analysis of food intake based on daily diaries.

#### 3.2.5. Antenatal and Postnatal Dietary Intake

The increased prevalence of allergic diseases over the last few decades may result from changes in prenatal or early-life environment, including maternal diet during pregnancy and dietary patterns during early childhood. Therefore, prevention of asthma by early allergen avoidance is essential in high-risk children, because early allergic sensitization is a significant risk factor for later development of asthma and atopic diseases [83]. Moreover, a recent review article suggested that antioxidant intake in prenatal and early life could protect against the development of atopic diseases [84]. Furthermore, recent studies of vitamin D intake suggest that it may be protective for atopy, although this still remains controversial [84]. In addition, early fish consumption could reduce significantly the risk of allergic diseases in children [85].

On the other hand, prolonged breastfeeding during the first year of life has been found to be protective against the development of atopy and asthma in children [86]. In contrast, a recent systematic review and meta-analysis showed that there was no association between breastfeeding and wheezing and asthma in children [87].

For this section, we reviewed in total 15 articles: seven review articles, one cross-sectional study, six cohort studies, and one systematic review and meta-analysis.

##### 3.2.5.1. Maternal Intake during Pregnancy

In the United Kingdom, Martindale *et al.* investigated maternal antioxidant intake during pregnancy and its relationship with asthma and atopic dermatitis [40]. There were no statistically significant associations between wheezing symptoms in the first two years of life and total maternal intake of β-carotene, selenium, magnesium, manganese, copper, and zinc. In the second year of life, there were significant negative associations with maternal total vitamin E intake (aOR = 0.49, 95% CI = 0.26–0.93) and maternal vitamin C intake was positively associated with wheezing (aOR = 3, 95% CI = 1.47–6.12) and eczema (aOR = 1.56, 95% CI = 0.99–2.45) [40]. An FFQ administered at 34 weeks gestation was used to assess diet during pregnancy. Despite its effectiveness to elicit accurate data on the dietary intake of foods containing antioxidant vitamins and minerals, FFQ estimates of dietary nutrient intake are dependent on subject recall of the foods consumed and of their quantities [24].

Previous studies that have investigated associations between dietary vitamin C, fruit, asthma, and respiratory symptoms have reported beneficial associations [52,54]. However, Martindale *et al.* unexpectedly found a positive association between maternal vitamin C intake and wheezing in children in the second year of life [40]. A possible explanation is that at high vitamin C concentrations might have a pro-oxidant effect, which could lead to respiratory symptoms [88]. Another possibility is that other added constituents of foods containing vitamin C might promote the development of wheezing in early life. Sulfites added as preservative to fruit drinks have been shown to cause wheezing in susceptible individuals [89]. No associations were found in that study between maternal dietary intake of selenium and wheezing or eczema in the first two years of life due to the reduced effectiveness of the FFQ in quantifying selenium intake because the correlation coefficient for selenium was weaker than that of vitamins E and C [40].

In Japan, Miyake *et al.* found that higher maternal consumption of green and yellow vegetables (aOR = 0.41, 95% CI = 0.24–0.71), citrus fruit (aOR = 0.53, 95% CI = 0.30–0.93), and β-carotene (aOR = 0.52, 95% CI = 0.30–0.89) during pregnancy may be protective against the development of eczema in infants [85]. Moreover, higher maternal vitamin E intake may reduce the risk of wheezing in infants (aOR = 0.54, 95% CI = 0.32–0.90). They also showed that higher maternal intake of total dairy products (aOR = 0.45, 95% CI = 0.25–0.79), milk (aOR = 0.5, 95% CI = 0.28–0.87), cheese (aOR = 0.51, 95% CI= 0.31–0.85), and calcium (aOR = 0.57, 95% CI = 0.32–0.99) was negatively associated with infantile wheezing but not with eczema (aOR = 0.54, 95% CI = 0.32–0.90). In contrast, higher maternal vitamin D intake might reduce childhood wheezing (aOR= 0.64, 95% CI= 0.43–0.97) and eczema (aOR = 0.63, 95% CI = 0.41–0.98), and a higher consumption of calcium and dairy foods during pregnancy may reduce the risk of childhood wheezing [46,90]. Data on maternal intake during pregnancy was evaluated by a diet history questionnaire that could only approximate consumption, so this would bias the estimates of the association toward the null. The diet history questionnaire was also designed to assess only recent dietary intake, so the data collected should only be taken as suggestive owing to the changing dietary habits of pregnant women due to the nausea they may experience during pregnancy.

Furthermore, studies have shown that vitamin D has many beneficial effects other than bone maintenance. The effect of vitamin D on surfactant production and in lung maturation has been confirmed in human studies [67]. Vitamin D is known to affect the immune system and lung development and function in children, so it might be protective for asthma [91].

In 2007, Devereux *et al.* showed that maternal intake of vitamin D was negatively associated with wheezing in five-year-old children [92]. Camargo *et al.* found that maternal intake of vitamin D was negatively associated with recurrent wheezing in children aged three years but no association with eczema was evidenced [93].

##### 3.2.5.2. Dietary Patterns in Early Childhood

One of the major modifiable dietary environmental risk factors for childhood asthma is the lack of breastfeeding [94]. A prospective birth cohort study in West Australia showed that the risk of childhood asthma aged six years was significantly reduced if exclusive breastfeeding was continued for at least the first four months of life [95].

A systematic literature review showed that avoidance of cow’s milk protein prevents the development of wheezing in atopic children [96] and that breast-milk should be the food of choice for all babies, except in some high-risk babies in whom hydrolyzed milk formula helps reduce the risk of developing asthma [97]. Moreover, frequent consumption of products containing milk fat in pre-school children was associated with a reduced risk of asthma symptoms [98]. A recent review article concluded that exclusive breastfeeding for four to six months and the postponement of solid food until four months of age are the main measures considered effective in the prevention of allergy in children [99].

## 4. Conclusions

In conclusion, various methodologies have been used to evaluate dietary factors in relation with asthma, allergic rhinitis and atopic dermatitis. Although the association of diet with allergic diseases has been studied worldwide, their findings are contradictory and sometimes barely significant. While the methods are all effective and useful in determining dietary factors, they seem less specific and sensitive for evaluating the quality of diet in childhood. Moreover, a child’s diet is not based on a single food item but is rather a complex of nutrients eaten together, which involves food interactions. For these reasons, the most suitable methodology for evaluating diet is one that evaluates long-term and short-term evaluation of dietary intake plus one to assess dietary patterns and quality of diet in relation with allergic diseases. Therefore, validation methods for assessing dietary habits, especially in epidemiology, should be developed because of it is not possible to use biological samples and biomarkers in all studies due to their high costs.

Moreover, we cannot exclude the possibility of a selection bias regarding the papers chosen to be included in this review; this is one limitation of our work. Hence, we suggested a future systematic and exhaustive review to be done on this large domain that would include all articles that dealt with diet and allergies. However, even if we can’t draw any final and definite conclusion based on this review, recommendations can be made on the main results that we have obtained from very large studies included in this work that could have a positive effect on limiting the rising of allergies in our time.

Despite these limitations, a major finding is that diet and allergic diseases are reality after all, they are not some myth or rumor that we are talking about more recently. Dietary factors may be an etiology of atopic diseases, especially in children. A healthy diet rich in antioxidants and omega-3 fatty acids consumed by the mother during pregnancy and by the child during childhood may reduce significantly the prevalence and incidence of asthma, allergic rhinitis, and atopic dermatitis, even in children with a hereditary predisposition for atopy. More prospective and interventional studies need to be performed on food groups and food items rather than on single nutrients to support the available results and widen the possibility of making generalizations. This could lead to recommendations for a special diet to be followed during pregnancy and in childhood, at least until adolescence. The “allergy epidemic” that the world is currently undergoing might thus be reduced.
